# Transgender EU Citizens and the Limited Form of Union Citizenship available to them

**DOI:** 10.1007/s10691-022-09491-9

**Published:** 2022-07-15

**Authors:** Serhii Lashyn

**Affiliations:** Universität Hamburg, Rothenbaumchaussee 33, 20148 Hamburg, Germany

**Keywords:** EU citizenship, Free movement, Transgender, Gender, Discrimination

## Abstract

This article argues that only a limited form of EU citizenship is available to transgender people. As the paper demonstrates, transgender Union citizens face numerous difficulties when they exercise their right to free movement, despite such movement being the core of Union citizenship. Rather, transgender individuals only have access to a considerably restricted form of EU citizenship which is guaranteed as part of their fundamental status conferred by EU Treaties. The article points out that the current approach of including transgender discrimination in the purview of discrimination on the grounds of sex fails to tackle the issue because it is conceptually problematic and largely ineffective. As a solution to remedy the issue, the paper suggests overhauling the existent approach to trans discrimination in a way that makes it capable of ensuring that transgender persons enjoy their Union citizenship to its fullest extent.

## Introduction

Think of a transgender man who is a national of a European Union (EU) Member State and raises two beautiful children. Having won a lengthy and exhausting legal fight with his own State, this man’s gender is now officially recognised. He now has new identity documents with a new name which reflect his gender correctly. However, this man is absolutely unable to change the birth certificates of his children, which indicate his old name and the sex he was assigned at birth. On those grounds, the authorities refuse to issue his children with travel documents. If asked by a police officer or border guard, this man will experience substantial difficulties proving that he is the parent. Because of that, he cannot travel with the kids for years. Despite the freedom of movement guaranteed by his “fundamental status”^1^, this man is confined to the borders of one country having done nothing wrong.

This is not a made-up example showing an improbable theoretical pitfall. This is the life story of Yanis, a 37-year-old trans man from Romania (Karsay 2021, 22–23). The story of Yanis is, in fact, not an isolated case. Across the EU, transgender people are afraid of travelling, as they know there will be issues with identity checks, let alone uncomfortable questions and suspicious looks; they do not start transitioning as they will have to divorce, they have to return back to their Member States because of bureaucratic obstructionism. Stories like this are rarely heard, but they attest to the fact that EU citizenship is substantially limited for transgender people. If EU law really “precludes national measures which have the effect of depriving citizens of the Union of the genuine enjoyment of the substance of the rights conferred by virtue of their status as citizens of the Union”^2^, why are transgender Union citizens treated so badly?

This article argues that only a limited form of EU citizenship is available to transgender people. The paper first demonstrates how transgender Union citizens systemically face some substantial hurdles while exercising their right to free movement. After that, it explores how the current approach to transgender discrimination is conceptually flawed. Finally, the article proposes an alternative approach to trans discrimination that has the potential of addressing the problems of transgender EU citizens in a more specific and, most importantly, effective manner.

Before turning to the core of this paper’s analysis, it is important to delimit the scope of one of the central terms used in this article. The word ‘transgender’ is used in this article in the meaning defined in Appendix A to the APA Guidelines for Psychological Practice with Transgender and Gender Nonconforming People, namely as “an umbrella term used to describe the full range of people whose gender identity and/or gender role do not conform to what is typically associated with their sex assigned at birth” (APA TGNC Guidelines 2015, 863). As the quoted passage demonstrates, this particular definition does not include intersex people, which is a topic that requires specific attention.

## How transgender people struggle to move freely in the EU

Neither of the core legal instruments of the EU protects transgender people from discrimination explicitly and directly. According to the Treaties, non-discrimination and tolerance are the values that prevail in all Member States.^3^ Furthermore, the Union guarantees that “[e]veryone is equal before the law”.^4^ As to the secondary law, the Equality Framework Directive does not mention gender identity as one of the grounds for discrimination that the Directive seeks to combat.^5^ Nevertheless, one should remember that the EU “is founded on the values of respect for human dignity, … equality, … and respect for human rights”.^6^ All these give grounds to conclude that, although not mentioned specifically, transgender individuals have the right to equality and protection against discrimination under EU law.

However, despite the solemn and promising language of the Treaties, surveys show that more than half of transgender persons experience discrimination in the EU (FRA Transgender Survey 2014, 21; FRA LGBTI Survey 2020, 22). Transgender discrimination on its own is a voluminous issue, but this article confines its analysis to and how EU law responds to this challenge. Specifically, I look into how transgender EU citizens face substantial difficulties in enjoying their EU citizenship status and how the current conceptual approach to transgender discrimination fails to address those particular difficulties.

EU citizenship may be briefly described as “a regionally-framed supranational citizenship” (Bosniak 2000, 457) of the EU. The nascent forms of common citizenship may be traced back to the Treaties of Paris (1951) and Rome (1957). Formally introduced in the Treaty of Maastricht in 1992, EU citizenship is a legal status that is common to the nationals of EU Member States and which is based on the direct connection between an individual and the Union. The status of EU citizen gives its holder a number of rights: freedom of movement and residence across the EU, the right to vote and stand as a candidate in the European Parliament elections and in the municipal elections of the Member State of residence, diplomatic protection and consular assistance when outside of the Union, the right to petition the European Parliament, access to the documents of the bodies of the EU, and the rights associated with taking part in European Citizens’ Initiative.^7^ As per the language of the Treaties, only the nationals of EU Member States are EU citizens, as the status is derived from the nationality of a Member State. Being a trailblazing and innovative concept in many ways, EU citizenship is nonetheless validly criticised for its exclusion of third-country nationals resident in the EU, a poorly functioning social dimension, and the disproportionate expansion of the scope of EU law (Lashyn 2021, 364–366).

It should be emphasised that freedom of movement was in place long before the Maastricht Treaty as the precursor of EU citizenship in its contemporary form (Jacobs 2007, 592–593) and still remains the central element of the concept of Union citizenship (Elsmore & Starup 2007, 70–72). Of course, EU citizenship goes far beyond free movement. Apart from the rights enumerated above, the status is linked to a set of fundamental rights (Van den Brink 2018, 22–26). It is still a contentious issue whether there are any duties attached to Union citizenship and, if there are some, what those are (Kochenov 2014; Bellamy 2015). Thus, the scope of EU citizenship is relatively wide and its precise delimitation requires some substantial analysis. When looking into how transgender people are hindered from enjoying their EU citizenship status, this paper focuses only on the freedom of movement. Apart from a rather utilitarian necessity to reasonably narrow the focal point of the article, there are other reasons for doing this. First, free movement remains the most articulated and developed part of EU citizenship. Other rights, such as those related to political participation, do not present significant difficulties for transgender EU citizens or, as in the case of diplomatic protection and consular assistance, are not detailed enough. Second, despite an ostensible tendency to gradually widen its scope, such as in the cases of *Tjebbes*^8^ or *D’Hoop*^9^, where the matters were between the state and its own nationals issues surrounding EU citizenship usually only arise when its holder moves to another Member State. The cross-border element triggers EU citizenship; therefore, this paper focuses precisely on the situations where transgender EU citizens move within the EU.

Formally speaking, transgender EU citizens are equally entitled to the freedom of movement and are equal to their fellow EU citizens in every aspect. However, the harsh realities make the exercise of this right quite difficult. Without claiming to be a comprehensive classification of this yet-to-be-studied facet of the freedom of movement, this article enumerates below three major types of such situations.

## Identity documents and identity checks

Identity verification is an essential component of one’s daily life (Köhler & Ehrt 2016, 8; Dunne 2017, 558; Lau 2020, 194). Showing an ID is necessary to access healthcare, book a hotel room, purchase an event ticket, and, very recently, prove one’s COVID-19 vaccination status before entering virtually any indoor public space. Despite their omnipresent inclusion in identity documents, Holning Lau rightfully describes gender markers as a “crude tool” for the purposes of verifying one’s identity (Lau 2020, 200–201). Pointing how important gender recognition is for individual well-being and self-perception, Florence Ashley questions the very necessity of including a gender marker in identity documents (Ashley 2021, 40–43). While this position is well-reasoned and it is true that identity verification can be done in a reliable way without checking the gender marker on documents, nowadays it is very difficult to imagine a world without the ubiquitous ‘M’ (male) and ‘F’ (female) in IDs.

Apart from the important role identity documents play in daily life, they are also a fundamental element of the freedom of movement in the EU. Identity documents are, in fact, the gatekeepers of cross-border mobility for EU citizens. Having “a valid identity card or passport”^10^ is a prerequisite for any form of cross-border movement inside the EU.^11^ Further, identity documents are needed for the issuance of registration certificates.^12^ If not discriminating on the basis of nationality, Member States are allowed to carry out identity checks as much as they choose to.^13^ On a broader note, identity documents are also considered one of the primary tools employed by states for distinguishing between residents and non-residents and differentiating between them in rights and obligations (Bruzelius 2019, 73). Such a differentiation particularly concerns voting rights, access to civil service, military obligations, and some other areas.

However, what goes unnoticed and appears natural to many can be a great challenge to others. In reality, getting the right identity documents and going smoothly through identity checks pose a great challenge to transgender persons. The most obvious problem is the difficulties that transgender people face when trying to obtain identity documents that display their gender correctly. The conditions for the legal recognition of one’s gender vary significantly across the Union. Only a handful of EU Member States allows gender recognition by means of simple self-declaration (European Commission, 2020, 111; Lau 2020, 205) which is considered to be the most appropriate option among others. This is confirmed by Principle 3 of Yogyakarta Principles (Yogyakarta Principles 2007) despite some criticism (Nirta 2021) and the fact that Yogyakarta Principles are, of course, neither a binding document nor part of EU law. The so-called depathologisation of transgender individuals has been recognised as the prevailing trend in the direction of a more humane approach to the issue (Cannoot 2019, 16–18). Despite this, the majority of EU countries still require transgender individuals to go through invasive procedures, such as surgery, hormone therapy, and even sterilisation (Dunne 2017, 558–560; European Commission, 2020, 112). Some States do not have a set of clear rules at all, thus throwing trans persons into the limbo of uncertainty and administrative arbitrariness (European Commission 2020, 114). In the cross-border context, the situation is getting much worse. While residing in one Member State, transgender persons are often forced to return to the Member State of their nationality in order to change their passports or residence cards after transitioning (Köhler & Ehrt 2016, 18). Furthermore, the availability of legal gender recognition procedures is sometimes contingent upon residence in the country (Scherpe & Dunne 2015, 629–631) or even citizenship status (European Commission 2020, 125–126). At the same time, even if legal gender recognition is available without unnecessary complications, it still may detrimentally affect one’s immigration status. For example, one trans woman from the Netherlands reported that she was effectively forced to refrain from gender transition because there was a risk that the six years of her previous residence will be lost for the purposes of naturalisation due to how this period of time is detailed in the identity documents (European Commission 2020, 126).

Apart from getting the right documents and managing legal gender recognition in the cross-border context, another problem for transgender EU citizens is going through checks and presenting the documents that they already have. Unable to obtain the documents which display their gender correctly, trans individuals face uncomfortable situations when their appearance does not match the gender marker on their passport or ID card according to the view of the border guard officer (Karsay 2021, 10). Gender stereotyping and non-conformity becomes an additional hurdle on the way of transgender EU citizens while travelling. When air travel becomes an embarrassing and even humiliating exercise, one may barely speak of the freedom of movement. All across the EU, even in the countries with progressive policies, transgender individuals feel discriminated against and embarrassed whenever they find themselves in situations where presenting their identity documents is necessary and those documents do not reflect their gender correctly (EU FRA 2014, 81–82). This certainly becomes a factor that discourages transgender EU citizens from enjoying freedom of movement in the EU.

## Family and children

The area of family life and children is a highly troubling matter for transgender people which presents a number of challenges, bureaucratic hurdles, professional mishandling, and social bias. In the context of crossing the inner borders of the EU, this area includes some more specific problems. In particular, transgender EU citizens face challenges related to their family members whose status often depends on the legal connection with them, such as their spouses or children. In this regard, one of the most disturbing practices is the so-called ‘forced divorces’. In the Member States with no marriage equality, married transgender persons are usually obliged to divorce before their gender transition may be legally recognised (Köhler & Ehrt 2016, 27–28; European Commission 2020, 161; Mills 2020, 270). The reason for this requirement is straightforward: allowing transgender individuals to remain married after their legal gender recognition would lead to lawfully existing same-sex marital unions in countries where people of the same sex are not allowed to marry (Scherpe & Dunne 2015, 632). This disheartening practice involves countless repercussions for the persons whose marriage is forcefully terminated by the State, of which high divorce-associated costs are often the least painful (European Commission 2020, 166).

From the viewpoint of human rights law, however, this practice is partially acceptable. In particular, in the *Hämäläinen* ruling, the European Court of Human Rights established that State authorities may require converting a marriage into a registered partnership as a prerequisite for legal gender recognition because there were “minor differences” between the two.^14^ However, even in the presence of an option to convert the marriage into a civil union or registered partnership with almost the same scope of rights, the compatibility of this requirement with the human rights standards remains doubtful, although it is acceptable under the case law of the Strasbourg Court. With regard to EU law, the Charter of Fundamental Rights guarantees every EU citizen “respect for his or her private and family life” (Article 7), “[t]he right to marry and the right to found a family” (Article 9) and proclaims that “[t]he family shall enjoy legal, economic and social protection” (Article 33(1)). These provisions give grounds to assume that requiring a divorce for legal gender recognition goes against the fundamental rights of transgender EU citizens even without a cross-border element.

This practice brings major complications for those Union citizens who have exercised their right to free movement. Some EU Member States require transgender persons to divorce for their status to be properly recognised but, unlike Finland, do not offer them an opportunity of partnership (European Commission 2020, 161). If the spouse of a transgender EU citizen was given the right of residence as a family member “accompanying or joining a Union citizen”^15^ who works in that EU Member State, such a divorce potentially threatens them with expulsion. The retention of their residence right becomes subject to the requirement of economic activity or financial self-sufficiency.^16^ As a result, for the sake of legal gender recognition, transgender individuals often have to destroy their family unions and risk being separated from their loved ones for an indefinite period of time. This practice is particularly appalling in light of the *Coman* judgment. In that decision, the Court established that Article 21(1) TFEU requires a Member State to extend a derived right of residence to the spouse of an EU citizen who came to that country because doing otherwise would discourage EU citizens from exercising their right to free movement.^17^ Furthermore, the Court specifically noted that the term “spouse” in this context is “gender-neutral”.^18^ When applying the *Coman* matrix to the situation of the spouses of transgender individuals, it is reasonable to conclude that, although the rules on a person’s status remain within Member States’ competence,^19^ this fact does not give enough grounds to upturn the family life of a EU citizen who availed themselves of free movement and went through legal gender recognition.

Another issue for transgender EU citizens is cross-border movement while accompanying their children. Apart from Yanis, whose story has been reported at the opening of this article, there are many more transgender parents who struggle to travel across the EU with their kids. For example, this is the case for those transgender parents living in one Member State but who are required to travel back to the Member State of their nationality for the purposes of legal gender recognition with their minor children who cannot be left at home (Karsay 2021, 13–14). While such travels may be humiliating because of the treatment by border guards, a problem that is more painful and much harder to resolve is the inability of transgender individuals to get properly registered as a parent (or, if they wish so, as a mother or father) on the birth certificates of their children (Köhler & Ehrt 2016, 33–34). Authorities usually refuse to correct birth certificates after either of the parents change their gender legally. As a consequence, it is quite hard for transgender persons to prove that they are parents when applying for a child’s passport (identity card) or whenever their parenthood has to be verified.

In *V.M.A.*, the Court of Justice dealt with the refusal of the Bulgarian authorities to issue a birth certificate to a child born in a same-sex marriage.^20^ The child was born in Spain where the couple resided and was issued a birth certificate indicating both women as parents.^21^ The formal reasons were that the authorities were unable to establish who the mother of the child was, and indicating two women as parents would go counter to the public policy of Bulgaria.^22^ The Court of Justice established that Bulgaria was obliged to issue the birth certificate where both women were indicated as parents.^23^ Citing *Coman*, the Court noted that those EU citizens who have exercised their right to free movement may rely on their rights pertaining to EU citizenship.^24^ Although the child has not exercised that right, she can invoke the rights associated with EU citizenship because her parents moved within the EU.^25^ Accordingly, the Bulgarian authorities are obliged to issue an identity card to the child regardless of the national provisions on birth certificates.^26^ In addition, Bulgaria has to issue a document that enables the child’s parents to accompany her while travelling.^27^ The Luxembourg Court emphasised that the matters of marriage and civil status remain within the Member States’ competence, but they must be regulated in compliance with the EU law on the freedom of movement.^28^ Furthermore, recognising the parental relationship established in another Member State does not interfere with the national identity of Bulgaria or its public policy.^29^

Although the judgment is certainly a positive development for same-sex parents in the EU, it has some substantial limitations which make the prospects of having such a precedent for transgender parents vague. First and foremost, the ruling was based heavily on the right to free movement. Although the right to family life as guaranteed by the Charter of Fundamental Rights was mentioned in the reasoning,^30^ the Court guarded that right only in so far as it is necessary for the child to exercise their right to free movement.^31^ Second, as follows from the preceding point, the Court did not require to recognise both women as the parents of the child but rather obliged Bulgaria to issue an identity card for her and to provide “a document which mentions them as being persons entitled to travel with that child”.^32^ For transgender EU citizens, these two limitations mean that they cannot rely on the judgment to demand corrections to the birth certificates of their children and, in absence of the cross-border element, they are not able to invoke their EU citizenship rights in order to exercise their right to free movement in the future.

## Social protection

While cross-border access to social assistance remains a problematic area of EU citizenship law in general (Bruzelius 2019), the issue gets more complicated for transgender EU citizens. One of the problems in that regard is the coverage of the costs associated with gender confirmation treatment which is often an expensive procedure. Although EU citizens are guaranteed equal access “to preventive health care and the right to benefit from medical treatment”,^33^ this is often not the case for transgender people. Across the EU, more than half of transgender individuals have to pay for their procedures exclusively from their own pockets (European Commission 2020, 168). In some Member States, it is virtually impossible to fund one’s gender transition by means of healthcare insurance (European Commission 2020, 168–169). The policies vary considerably across the EU. Some Member States make funds available only for some parts of gender confirmation treatment, while some Member States reimburse less than others (Van den Brink & Dunne 2018, 79). Almost everywhere in the EU, transgender citizens face serious financial hurdles on the way to transitioning.

For those EU citizens who move from their home country to another Member State, funding their treatment presents an additional challenge. Even if they work and pay all contributions, it may be impossible for EU citizens to get gender confirmation procedures covered by the healthcare plan depending on which EU Member State they reside in. EU citizens coming from some progressive Member States to a more conservative environment are often unable to receive the coverage they can expect in their home country. As a consequence, those EU citizens have to travel back (facing the problems connected with identity documents and travels with children as described above), pay much more out of their pockets, or even delay gender transition for an indefinite period of time. The cross-border element presents one more problem: When EU citizens go to another EU country for their gender confirmation treatment because of more affordable prices or better conditions, it is often the case that their procedure is not recognised in their home Member State which, in its turn, is responsible for the legal formalities of gender recognition (Van den Brink & Dunne 2018, 79). While free movement allows EU citizens to seek lower prices, it also makes legal gender recognition a very difficult task, especially for those transgender persons who come from EU countries with restrictive policies on civil status.

All these show that the current state of affairs, from the viewpoint of equal access to healthcare, discourages transgender EU citizens to make use of their right to free movement or even punishes them for doing that. Access to the social systems of host Member States for immigrant EU citizens remains to be substantially hindered in many EU countries. It is certainly true that new community members may legitimately expect some support only after they have been contributing their fair share to support other members for some time (Kramer 2016, 273–278). This model is de facto implemented in the Citizenship Directive and in the jurisprudence of the Court of Justice (Mantu & Minderhoud 2017, 706–709). However, a striking injustice is done to those transgender EU citizens who actively contribute to the social systems of their host Member States and diligently pay healthcare insurance premiums but do not get expected support when they need it. Although it is settled in the case law of the Court of Justice that Member States have to ensure that EU citizens are treated in the same manner as their own nationals,^34^ it is also true that the financial hurdles that are capable of making gender confirmation treatment inaccessible in some Member States interfere with freedom of movement in a way that is prohibitive for transgender EU citizens.

## The failing approach to transgender discrimination in EU law

What kind of protection against transgender discrimination does EU law offer? Although the above remarks clearly indicate that, whatever it is, the approach adopted by the EU law is certainly failing, it is worthy of the effort to look into this approach carefully in order to identify why it is ineffective for transgender EU citizens and prepare some base for offering a conceptually better alternative.

The foundation of the stance that EU law takes on transgender discrimination was laid down in the case of *Cornwall City Council*. The case was brought by an educational manager who was dismissed from her establishment after going through gender transition, allegedly due to redundancy.^35^ In fact, the dismissal was motivated by gender reassignment.^36^ The competent tribunal decided that the case did not fall within the scope of the legal provisions on sex discrimination as the employee would have been dismissed in case of a gender transition regardless of the sex assigned at birth.^37^ The Court of Justice was called upon to decide whether the relevant provisions of EU law prohibit such a dismissal.^38^ The Court noted that the prohibition of sex discrimination “is simply the expression, in the relevant field, of the principle of equality”.^39^ Without offering an elaborated explanation, the Court stated that, as “one of the fundamental human rights”,^40^ protection against discrimination on the grounds of sex includes the situations of those people who underwent gender reassignment.^41^

The rationale offered by the Luxembourg Court is vulnerable to critique. The ruling itself admits that the applicant was treated unfavourably in comparison with the individuals “of the sex to which he or she was deemed to belong before undergoing gender reassignment”.^42^ Rephrased in a more accessible way, the Court’s ruling points to the fact that the applicant was discriminated in comparison not with people of the opposite sex but with cisgender people. This reveals the fundamental flaw of the approach taken by EU law: the law treats transgender discrimination as sex discrimination. A careful reading of the Court’s own explanation demonstrates that the applicant was discriminated not on the grounds of sex but on the grounds of gender identity. The applicant would not have been dismissed if they did not embark on gender transition. This flawed approach is also the reason why the decision of the Court did not respond to the arguments offered by the UK which claimed that, although the applicant was dismissed after transitioning from male to female, the same would have happened to someone transitioning from female to male and thus a transgender man would have been treated unfavourably in the very same manner as a transgender woman.^43^ Indeed, treating both transgender women and transgender men less favourably than others makes accusations of sex discrimination logically incoherent. It is regrettable that the Court avoided addressing this line of argumentation.

The same approach to transgender discrimination was later reconfirmed in *K.B.*, *Richards*, and *MB*. All three cases originated from the UK and concerned pensions. *K.B.* concerned a woman who was employed by the National Health Service (NHS) and lived in a family with a transgender man.^44^ As her long-term partner was not able to legally recognise his gender, the applicant could not marry him and thus he was not entitled to the survivor’s pension under the NHS scheme.^45^ The Court deemed the scheme discriminatory on the grounds of sex and in comparison to those couples “where neither partner's identity is the result of gender reassignment surgery”.^46^ In *Richards*, the case was brought by a transgender woman who has been refused earlier retirement on the grounds that she was legally deemed male.^47^ Despite the fact that the Member States are expressly allowed to differentiate between men and women in the domain of pensions,^48^ the Court found that the law was discriminating on the grounds of sex between transgender women and those “women whose gender is not the result of gender reassignment surgery”.^49^ Although the case followed the established line of treating transgender discrimination as a particular case of sex discrimination, the wording of the decision, such as the quote above, clearly indicates that, in fact, discrimination was done between cisgender women and transgender women, not between men and women. Finally, the case of *MB* concerned a married transgender woman who, however, could not get her gender legally recognised due to the requirement of forced divorce and her unwillingness to terminate the marital union due to her religious views.^50^ As she was deemed legally male, the applicant could not retire earlier.^51^ The Court found that the UK law discriminates on the grounds of sex^52^ but, maintaining the familiar approach to transgender discrimination, stated that the law “treats less favourably a person who has changed gender after marrying than it treats a person who has retained his or her birth gender and is married”.^53^

The relevant jurisprudence of the Court of Justice subsumes transgender discrimination under the category of sex discrimination, the prohibition of which, in its turn, flows from the foundational principle of equality enshrined in the primary law of the EU. However, the facts of the cited cases clearly demonstrate that transgender Union citizens were treated less favourably in comparison with cisgender persons, meaning those people whose gender feeling matches the sex assigned at birth and who have not gone through gender transitioning.

## Gender identity as grounds for discrimination

Before introducing a more coherent way to treat discrimination against transgender EU citizens, this article demonstrates exactly why the current approach is flawed. There are two main points of critique.

The first is that the current approach to discrimination against transgender EU citizens is simply logically wrong. The theoretical discourse on discrimination always includes, among others, the following two elements: the grounds for discrimination and a comparison of the treatment of the person asserting the discrimination with someone who has not suffered the discrimination complained of (Moreau 2010; Segall 2012; Lippert-Rasmussen 2015). The jurisprudence of the Court of Justice acknowledges the fact that transgender EU citizens are treated less favourably than cisgender citizens but classifies it as a case of sex discrimination. In reality, transgender individuals are discriminated against not on the grounds of sex and in comparison with people of the opposite sex but on the grounds of their gender identity and in comparison with cisgender people. Sex discrimination and discrimination on the basis of gender identity are two separate grounds and should be treated as such.

Second, the current approach is ineffective. Although it helped to protect transgender individuals in the cases cited above, its containment to the domain of sex discrimination imposes some substantial limits on its potency. The current approach is conceptually unable to address effectively the situations where drawing a link to sex discrimination is difficult and where there is a need for specific measures aimed at protecting transgender EU citizens, such as in the case of free movement. In order to ensure that the fundamental rights of transgender persons and the rights conferred upon them by the Treaties are respected, transgender discrimination should be separated into its own category. To illustrate the ineffectiveness of the current approach, it is useful to note that the Court stated consistently that it is up to the Member States to decide on the conditions for legal gender recognition.^54^ The only limitation is adherence to the principle of non-discrimination^55^ which means that everyone should have equal access to such procedures, however difficult or burdensome they might be. Therefore, as long as EU citizens of both female and male sexes have equal access to legal gender recognition or are equally precluded from it, EU law is of little help.

One may speculate about the reasons for the adoption of this approach. Holning Lau suggests that, in general, the inclusion of transgender discrimination in the domain of sex discrimination may be explained in the following way. First, transgender people challenge the sex stereotypes prevalent in the given society and thus their discrimination is based on sex (Lau 2018, 10). This is also called gender non-conformity theory (Koch & Bales 2008, 249). Second, when someone is being treated less favourably for transitioning from being a man to being a woman, it means discrimination based on sex (Lau 2018, 10–11). Lau suggests that the Court of Justice was guided by the transitioning argument when subsuming transgender discrimination under the category of discrimination on the grounds of sex (Lau 2018, 10–11). This theoretical explanation, however, does not stand. First and foremost, it equates sex to gender. This is obviously not the case (Torgrimson & Minson 2005). As some commentators rightly point out, “sex seems to be a poor proxy for gender, because it is incorrect to assume that sex precedes and determines gender” (Lindqvist et al. 2021, 334). Second, as Lau points out himself, there is the counterargument of the symmetry defence. The symmetry defence argues that there is no discrimination based on sex if it is done equally to transgender men and transgender women (Lau 2018, 12–13). In other words, if both trans men and trans women are treated less favourably than cisgender men and cisgender women, there is no sex discrimination. As mentioned above, the Court of Justice failed to address this counterargument and nevertheless subsumed transgender discrimination under the umbrella of sex discrimination.

It may be suggested that the approach employed by the Court was a choice in favour of a simpler and easily accessible tool when there was a need to protect a particularly vulnerable group. It was done not in a far-sighted manner but certainly with the best intentions in mind. In 1996, when *Cornwall County Council* was decided, the social attitudes across the EU were quite different: there was still no country where same-sex marriage was legal. When faced with the difficult task of shielding transgender people from discrimination by means of EU law, the Luxembourg judges opted for an alternative that seemed easier than others, less revolutionary, and a bit straightforward. The societal demands have changed significantly since then. Something that worked well in the past does not work anymore.

Under the current approach, a decision akin to *Coman* in the area of transgender discrimination is not feasible. The *Coman* case concerned the American husband of a man with American and Romanian nationalities who was refused residency in Romania on the grounds that the Romanian law did not recognise same-sex marriages.^56^ The Court of Justice declared that the term ‘spouse’ used in the Citizenship Directive is ‘gender-neutral’^57^ and the husband of the applicant must be allowed to reside in Romania as doing otherwise would undermine the right to the freedom of movement conferred upon Mr. Coman by the Treaties.^58^

Can the spouse of a transgender EU citizen, forced to divorce due to their spouse’s gender transition and thus threatened with potential expulsion, anticipate the same protection from EU law as the husband of Mr. Coman got? Apparently, not. While *Coman* was validly criticised for being limited only to EU citizens who find themselves in cross-border situations (Tryfonidou 2019, 219–220), transgender EU citizens cannot expect even this level of protection. The Court of Justice stated that the requirement of divorce is not incompatible with EU law.^59^ Therefore, there will be no opportunity to protect the family life of a transgender EU citizen under the category of sex discrimination. In the same manner, transgender EU citizens cannot expect a precedent like *V.M.A.* as that case was not decided on the basis of sex discrimination at all.

There is only one way to address the whole range of issues facing transgender EU citizens when they exercise their right to free movement in a coherent and effective way. This paper proposes approaching transgender discrimination as discrimination on the grounds of gender identity. Under the proposed approach, transgender EU citizens would be protected whenever and wherever they are treated less favourably than cisgender people. These grounds for discrimination would exist on an equal footing with other grounds, such as sexual orientation, sex, religion, age and so on. The conceptual difference between sex and gender identity is the cornerstone of this proposed approach. While birth assigned sex is generally understood as differentiation in humans based on their reproductive organs, gender identity is best described as “each person’s deeply felt internal and individual experience of gender, which may or may not correspond with the sex assigned at birth, including the personal sense of the body … and other expressions of gender, including dress, speech and mannerisms” (Yogyakarta Principles 2007, preamble). With this definition of gender identity in use, discrimination against transgender EU citizens will be treated as discrimination against those people whose gender identity does not correspond to the birth-assigned sex and not as discrimination against people with different reproductive organs. The logical coherence of this approach allows for better accommodation of the interests of transgender EU citizens and will enable effective remedying of the specific issues they face when exercising their right to free movement. While the regulation of the matters pertaining to civil status will further remain in the competence of the Member States, the proposed approach will prevent all Member States from using that competence in order to hinder transgender EU citizens’ access to the freedom of movement. In *Coman* and *V.M.A.*, Member States were prohibited from using their definition of marriage in a way that negatively affects the right to free movement while retaining their understanding of marriage as a union between persons of the opposite sex. The proposed approach does exactly the same with regard to transgender individuals.

The approach suggested above does not require treaty revisions and can be implemented in two ways. First, it may take place through a change of the attitude of the Court of Justice towards transgender discrimination. One may reasonably expect that there will be more cases pertaining to the difficulties transgender EU citizens face when moving within the EU. Challenged with the specific nature of transgender issues, the Court will eventually understand that its approach has to evolve conceptually. The second way is amendments to the secondary law and the adoption of new rules aimed at enhancing the protection of transgender EU citizens. This is less likely to happen as the Commission’s Strategy for 2020–2025 fails to take into account the particular problems of transgender EU citizens that arise while exercising their right to free movement. The Commission promises to “continue to ensure the correct application of free movement law, including to address specific difficulties preventing LGBTIQ people and their families from enjoying their rights” (European Commission 2020, 14). The respective part of the Strategy is quite superficial and is narrowed to further implementation of *Coman* and other facilitation of free movement for rainbow families (European Commission 2020, 14–15).

## Conclusions

As this contribution has demonstrated, the freedom of movement is significantly limited for transgender EU citizens. Unable to get identity documents that suit them, embarrassed to go through identity checks, denied correcting the birth certificates of their children, forced to divorce their loved ones, not being able to afford gender confirmation treatment in host Member States—transgender people all across the EU are discouraged to take advantage of the right conferred upon them by the Treaties and that forms their fundamental status as EU citizens. Despite the gradual development of the EU “from an economic to a political community” (Wollenschläger 2011, 30) and the pivotal role of EU citizenship in that process, the latter remains to be chiefly contained to the right to free movement. However, transgender EU citizens only have access to a substantially limited form of this right. This situation should be dealt with seriously.

It should be noted that this contribution focuses only on the cross-border issues facing transgender EU citizens. The problems that happen within national borders or the issues experienced by stateless persons or third-country nationals in the EU are left outside of the purview of this paper. And yet, even despite its limited and purposefully narrowed focus, this contribution reveals that the problems are substantial.

My analysis demonstrates that transgender EU citizens face numerous challenges while exercising their right to free movement. The scale of those challenges and their diversity allow speaking of only a significantly limited form of EU citizenship available to transgender EU citizens. One of the main reasons that such unfair treatment goes on is because of the problematic approach to transgender discrimination in EU law, which subsumes it under the category of sex discrimination. A new and better approach is needed. Such an alternative could be treating transgender discrimination as discrimination on the grounds of gender identity.

Gender and the freedom to express one’s gender identity are crucial to one’s self-perception and personality. Judging from their experience of frequent travels across the EU, a transgender person from Ireland described themselves as a “second-class citizen” (EU FRA 2014, 43). This is undoubtedly wrong. No EU citizens should feel inferior to others. Transgender persons deserve a fully functional form of EU citizenship, not a limited second-class version of it.

## Footnotes

1 *Rudy Grzelczyk v Centre public d’aide sociale d’Ottignies-Louvain-la-Neuve* [2001] Case C-184/99 ECLI:EU:C:2001:458, 2001 European Court Reports 6193, at para 31.

2 *Gerardo Ruiz Zambrano v Office national de l’emploi (ONEm)* [2011] Case C-34/09 ECLI:EU:C:2011:124, 2011 European Court Reports 1232, at para 42.

3 Consolidated version of the Treaty on European Union, OJ C326/13 (TEU), art 2.

4 Charter of Fundamental Rights of the European Union, OJ C326/391 (Charter of Fundamental Rights), art 20.

5 Directive 2000e/78/EC of 27 November 2000 Establishing a General Framework for Equal Treatment in Employment and Occupation, OJ L303/16, art 1.

6 TEU, art 2.

7 Consolidated version of the Treaty on the Functioning of the European Union, OJ C326/47 (TFEU), art 15(3), 20–24; Charter of Fundamental Rights, art 39, 40, 42–26.

8 *MG Tjebbes and Others v Minister van Buitenlandse Zaken* [2019] Case C-221/17 ECLI:EU:C:2019:189, 2019 Digital Reports of Cases.

9 *Marie-Nathalie D’Hoop v Office national de l’emploi* [2002] Case C-224/98 ECLI:EU:C:2002:432, 2002 European Court Reports 6191.

10 Directive 2004/38/EC of 29 April 2004 on the Right of Citizens of the Union and their Family Members to Move and Reside Freely within the Territory of the Member States, OJ L158/77 (Citizenship Directive), art 4.

11 *Supra* n 10, art 4-6.

12 *Supra* n 10, art 8(3).

13 *Supra* n 10, art 26.

14 *Hämäläinen v Finland*. 2014. Application no 37359/09 ECLI:CE:ECHR:2014:0716JUD003735909, at para 87.

15 Citizenship Directive, art 7(1)(d).

16 *Supra* n 15, art 13(1).

17 *Relu Adrian Coman and Others v Inspectoratul General pentru Imigrări and Ministerul Afacerilor Interne* [2018] Case C-673/16 ECLI:EU:C:2018:385, 2018 Digital Reports of Cases, para. 24.

18 *Supra* n 17, at para 35.

19 *Supra* n 17, at para 37.

20 *V.M.A. v Stolichna obshtina, rayon “Pancharevo”*. 2021. Case C-490/20 ECLI:EU:C:2021:1008, 2021 Digital Reports of Cases", paras. 18–23.

21 *Supra* n 20, at paras 18-19.

22 *Supra* n 20, para. 23.

23 *Supra* n 20, at para 69.

24 *Supra* n 20, at para 42.

25 *Supra* n 20, at para 42.

26 *Supra* n 20, at paras 44-46.

27 *Supra* n 20, at para 50.

28 *Supra* n 20, at para 52.

29 *Supra* n 20, at para 56.

30 *Supra* n 20, at paras 47, 59–64.

31 *Supra* n 20, at para 65.

32 *Supra* n 20, at para 50.

33 Charter of Fundamental Rights, art 35.

34 *D'Hoop*, at para 31.

35 *P v S and Cornwall County Council* [1996] Case C-13/94 ECLI:EU:C:1996:170, 1996 European Court Reports 2159, paras 3–5.

36 *Supra* n 35, at para 6.

37 *Supra* n 35, at para 7.

38 *Supra* n 35, at para 13.

39 *Supra* n 35, at para 18.

40 *Supra* n 35, at para 19.

41 *Supra* n 35, at para 20.

42 *Supra* n 35, at para 21

43 *Supra* n 35, at para 15.

44 *K.B. v National Health Service Pensions Agency and Secretary of State for Health* [2004] Case C-117/01 ECLI:EU:C:2004:7, 2004 European Court Reports 568, at paras 11–12.

45 *Supra* n 44, at paras 12-13.

46 *Supra* n 44, at para 31.

47 *Sarah Margaret Richards v Secretary of State for Work and Pensions* [2006] Case C-423/04 ECLI:EU:C:2006:256, 2006 European Court Reports 3602, at paras 14–16.

48 *Supra* n 47, at paras 25, 33.

49 *Supra* n 47, at para 29.

50 *MB v Secretary of State for Work and Pensions* [2018] Case C-451/16 ECLI:EU:C:2018:492, 2018 Digital Reports of Cases, at paras 16-17.

51 *Supra* n 50, at paras 18-19.

52 *Supra* n 50, at para 52.

53 *Supra* n 50, at para 37.

54 *Supra* n 50, at para 29;* Richards*, at para 21.

55 *MB*, at para 29.

56 *Coman*, at para 29.

57 *Supra* n 54, at para 35.

58 *Supra* n 54, at paras 40, 45, 51.

59 *Richards*, at para 29.
